# Software reliability model of open source software based on the decreasing trend of fault introduction

**DOI:** 10.1371/journal.pone.0267171

**Published:** 2022-05-02

**Authors:** Jinyong Wang, Ce Zhang, Jianying Yang

**Affiliations:** 1 School of Automation and Software Engineering, Shanxi University, Taiyuan, People’s Republic of China; 2 School of Computer Science and Technology, Harbin Institute of Technology at Weihai, Weihai, People’s Republic of China; Shandong University of Science and Technology, CHINA

## Abstract

Open source software (OSS) has become one of the modern software development methods. OSS is mainly developed by developers, volunteers, and users all over the world, but its reliability has been widely questioned. When OSS faults are detected, volunteers or users send them to developers by email or network. After the developer confirms the fault, it will be randomly assigned to the debugger who may be a developer, a volunteer, or a user. These open source community contributors also have the phenomenon of learning when removing faults. When the detected faults are removed, the number of introduced faults decreases gradually. Therefore, this study proposes a software reliability model with the decreasing trend of fault introduction in the process of OSS development and testing. The validity of the proposed model and the accuracy of estimating residual faults are verified by experiments. The proposed model can be used to evaluate the reliability and predict the remaining faults in the actual OSS development and testing process.

## I. Introduction

Recently, the rise of open source software (OSS) has become another way of development relative to closed source software. Since the development and testing process of OSS is mainly completed by developers, volunteers, and users all over the world, its reliability has been widely questioned. Although the current OSS adopts the strategy of “early release and frequent release” [1] to improve the reliability of software, the actual determination of OSS release is a difficult problem. Moreover, in this way, human and subjective factors are too strong, and this condition is not conducive to improving the reliability of the actual OSS.

Considering that volunteers’ or users’ interests change in the process of OSS development and testing, Li et al. [2] proposed an OSS reliability model with the fault detection rate increasing first and then decreasing. Wang and Mi [3] studied the trend of the decline in the fault detection rate during OSS development and testing, and they proposed a reliability model of OSS based on the decline in the fault detection rate. Wang [4] also proposed that fault introduction obeys a Pareto distribution and established the corresponding single release OSS reliability model. In addition to the abovementioned two single release OSS reliability models, Singh et al. [5] proposed a multi-release OSS reliability model by studying the entropy change of OSS source code. Tandon et al. [6] proposed the corresponding multi-release reliability models of OSS by studying the distribution of OSS in the process of fault detection.

Notably, the difference between single release OSS reliability model and multi-release OSS reliability model is that they are the two kinds of modeling ideas. The former assumes that the faults in each version of OSS are independent of each other [7]. The latter assumes that faults in the current version are related to faults in previous versions, and faults in the current version also affect fault detection and removal in later versions.

Although some of the abovementioned OSS reliability models can effectively evaluate the reliability of OSS under certain conditions, they ignore another phenomenon in the process of OSS development and testing, that is, the gradual decrease in the number of introduced faults over time. The reason why the number of introduced faults is decreasing is closely related to the learning phenomenon [8] of OSS debuggers and their hobbies, interests, and professional skills. In view of this situation, we propose an OSS reliability model based on fault introduction decline variation in this study. The validity and accuracy of the proposed model are verified by relevant experiments.

The contributions of this work are described as follows:

1. It is first to propose that the number of introduced faults is gradually decreasing in the process of OSS development and testing. The number of faults introduced is related to the learning process, professional knowledge, interests, and hobbies of debuggers.
2. The decreasing changes of fault introduction are consistent with the actual development and testing process of OSS.

The rest of the paper is organized as follows:

Section II explains the reason of the decreasing changes of fault introduction during OSS development and testing. Section III describes the developed process of the proposed model. Section IV deals with the model parameter estimation. Section V provides an experiment on the fitting and predictive performances of the proposed model compared with other models. Moreover, fault data sets of OSS are introduced, model comparison criteria are described, comparison models are provided, and the fitting and predictive performances of the proposed model are analyzed and discussed. Section VI gives the sensitivity analysis of model parameters. Threats to validity of the proposed model are presented in Section VII. Section VIII contains a review of the literature. Conclusions are drawn in Section IX.

## II. Reasons for the gradual reduction in fault introduction during OSS development and testing

We consider that, in the process of OSS development and testing, the reasons for the decline in fault introduction come from three aspects: first, learning factors [8]; second, interests and hobbies of developers or volunteers; third, the professional skills of developers or volunteers.

1. Learning factors
  After the OSS fault is sent to the developer through email or network, the developer first confirms whether it is a real fault, and then, it randomly assigns it to the corresponding debugger. When the debugger removes the fault, he also has a continuous rich experience and in-depth understanding of the corresponding OSS functions or products. With the current software development and testing, detected faults are becoming less, and the experience or knowledge of debuggers is also growing, that is, a learning process. Therefore, when detected faults are removed, the number of introduced faults will gradually become less. Fault introduction is decreasing with the development and testing of OSS.
2. Interests and hobbies of developers or volunteers
  These debuggers are also fans of OSS; they have a strong interest in the current development of OSS projects. These factors drive them to devote themselves wholeheartedly to determine the cause of the fault, try their best to remove the fault, and attempt to reduce the introduction of new faults.
3. Professional skills of developers or volunteers
  Debuggers themselves are experts in the field in general. They have deep insight, rich experience, and knowledge on the development of the software. When they remove faults, the probability of introducing new faults is very small. With the development and testing of OSS, they have a deep understanding of the function and role of the software. When they remove faults, the number of introduced faults will gradually become less.

## III. Reliability model of OSS based on gradual decrease in fault introduction

OSS fault detection usually follows nonhomogeneous Poisson process (NHPP). In other words, the fault detection process of OSS can be regarded as a counting process, which is represented by *N*(*x*). It is defined as follows:

$$\mathrm{Pr}\{N(x)=n\}=\frac{\xi (x)}{n!}\exp(-\xi (x)),\;n=0,1,2,\ldots ,k,$$ (1)

where Pr{} and *ξ*(*x*) represent a probability and mean value function, respectively. x denotes a random variable.

Assumptions of the proposed model are listed as follows:

1. Fault detection of OSS follows NHPP and the faults detected in OSS are removed immediately.
2. The number of faults detected in OSS is related to the number of remaining faults in the software.
3. New faults can be introduced when the faults in OSS are removed.
4. Debuggers have the phenomenon of learning in the process of fault removal in OSS.
5. The number of fault introduction is gradually decreasing in the process of OSS development and testing.

They can be obtained from assumptions 1 and 2,

$$\frac{d\xi (t)}{dt}=\psi (t)(\phi (t)-\xi (t))$$ (2)

and

ϕ(t)=ηF(t)+C (3)

Herein, *ξ*(*t*), *ψ*(*t*), and *ϕ*(*t*) represent the expected cumulative number of detected faults, the fault detection rate function, and the expected total number of initially detected and introduced faults by time *t*, respectively. *η*, *F*(*t*), and *C* denote the expected total number of initially introduced faults, the fault introduction rate function obeying a distribution, and the expected total number of initially detected faults, respectively.

It can be derived from Assumption 4,

$$\psi (t)=\frac{\psi }{1+\gamma \exp(-\psi t)}$$ (4)

where *ψ* and *γ* represent a fault detection rate and a fault inflection factor, respectively.

According to Assumptions 3 and 5, we give a distribution function considering fault introduction with a decreasing trend over time.

$$F(t)=1-\frac{\exp(-\mu t)}{1+\mu t},\;\mu >0$$ (5)

where *μ* is a scale parameter. *t* is a time variable.

Simultaneous solution of Eqs (2)–(5) is

$$\left\{ \begin{array}{l} \frac{d\xi (t)}{dt}=\psi (t)(\phi (t)-\xi (t))\\ \phi (t)=\eta F(t)+C\\ F(t)=1-\frac{\exp(-\mu t)}{1+\mu t}\\ \psi (t)=\frac{\psi }{1+\gamma \exp(-\psi t)} \end{array}\right.$$ (6)

The proposed model can be expressed mathematically as follows:

$$\xi (t)=\frac{\eta (1-\frac{\exp(-\mu t)}{1+\mu t})+C(1-\exp(-\psi t))}{1+\gamma \exp(-\psi t)}-\frac{\eta \mu \exp(-\psi t)\sum_{j=0}^{m}\sum_{i=0}^{n}\frac{{\left(-1\right)}^{j}(j+2)\mu^{j}{\left(\psi -\mu \right)}^{i}t^{i+j+1}}{(i+j+1)i!}}{1+\gamma \exp(-\psi t)}$$ (7)

Detailed solution processes can be found in Appendix A.

## IV. Model parameter estimation method

In this study, least square estimation (LSE) of nonlinear regression is used to estimate parameters’ values of OSS reliability models. It is defined as follows:

$$\Theta =\sum_{i=0}^{n}{\left(\xi (t_{i})-O_{t_{i}}\right)}^{2}$$ (8)

where $O_{t_{i}}$ represents the number of observed faults by time *t*_*i*_.

When taking partial differential on both sides of Eq (8),

$$\left\{ \begin{array}{l} \frac{\partial \Theta }{\partial \eta }=\sum_{i=0}^{n}\frac{\partial (\xi (t_{i}))}{\partial \eta }(\xi (t_{i})-O_{t_{i}})=0\\ \frac{\partial \Theta }{\partial C}=\sum_{i=0}^{n}\frac{\partial (\xi (t_{i}))}{\partial C}(\xi (t_{i})-O_{t_{i}})=0\\ \frac{\partial \Theta }{\partial \psi }=\sum_{i=0}^{n}\frac{\partial (\xi (t_{i}))}{\partial \psi }(\xi (t_{i})-O_{t_{i}})=0\\ \frac{\partial \Theta }{\partial \mu }=\sum_{i=0}^{n}\frac{\partial (\xi (t_{i}))}{\partial \mu }(\xi (t_{i})-O_{t_{i}})=0\\ \frac{\partial \Theta }{\partial \gamma }=\sum_{i=0}^{n}\frac{\partial (\xi (t_{i}))}{\partial \gamma }(\xi (t_{i})-O_{t_{i}})=0 \end{array}\right.$$ (9)

By solving the Eq (9) simultaneously, we can obtain the estimated values of parameters of the proposed model. Notably, we use LSE to estimate parameters’ values of models instead of maximum likelihood estimation (MSE). Some cases may have no maximum likelihood estimation function value. Therefore, the parameters’ values of the model cannot be estimated. It is not conducive to the performance comparison of the model.

## V. Numerical examples

### A. OSS fault data sets

We use two fault data sets collected from Apache Storm and Apache Chemistry OpenCMIS projects of OSS products in issue tracking systems (https://issues.apache.org/) to verify the fitting and predictive performances of the proposed model. Apache Storm is a distribution system of real-time computation with free and open source nature. It can deal with unbounded data streams and batch processing like Hadoop, but it is much easier to use with any programming language. Apache Chemistry OpenCMIS simplifies the client and server development of content management interoperability services, and it provides API, SPI, and testing tools and allows content server and application developers to focus on ECM domain model without relating to the underlying communication rules.

The first fault data sets (DS1) from Apache Storm project include three releases, namely, STORM 1.0.1 (Release-1), STORM 1.0.2 (Release-2), and STORM 1.0.3 (Release-3), which are denoted as DS1-1, DS1-2, and DS1-3. The second fault data sets (DS2) from Apache Chemistry OpenCMIS project include three releases, namely, OpenCMIS 0.4.0 (Release-1), OpenCMIS 0.5.0 (Release-2), and OpenCMIS 0.6.0 (Release-3), which are denoted as DS2-1, DS2-2, and DS2-3. We use the first fault data sets (DS1) to compare the goodness-of-fit of the proposed model with those of other models. In addition, we use the second fault data sets (DS2) to validate the prediction performance of the proposed model.

For DS1-1, 33 faults are collected using 32 months from December 2013 to July 2016. For DS1-2, 61 faults are obtained consuming 17 months from March 2015 to July 2016. DS1-3 collects 70 faults using 71 weeks from November 2015 to March 2017. In DS2-1, 40 faults are obtained using 65 weeks from March 2010 to June 2011. In DS2-2, 20 faults are collected using 31 weeks from May 2011 to November 2011. In DS2-3, 31 faults are detected using 16 weeks from August 2011 to December 2011.

In issue tracking systems, each issue (fault) has multiple attributes, and each attribute has multiple sub-attributes. For example, the attribute of the issue (fault) mainly includes Type, Key, Summary, Assignee, Reporter, Priority, Status, Resolution, Created and Updated. Moreover, the sub-attribute of Status mainly includes OPEN, REOPENED, RESOLVED, and CLOSED. The sub-attribute of Resolution has Unresolved, Fixed, Duplicate, Not A Problem, Cannot Reproduce, and Would Not Be Fixed. Notably, fault data sets in this paper are collected from fixed version excluding the sub-attributes of faults (issues) with Unresolved, Duplicate, Not A Problem, Cannot Reproduce, and Would Not Be Fixed. We select fault data sets of OSS randomly to test the adaptability, stability, and robustness of the proposed model.

### B. OSS comparison models and criteria

In this study, we use five OSS reliability models to compare with the proposed model. The five OSS reliability models include two types: one is a single release OSS reliability model, and the other is a multi-release OSS reliability model. For example, the Li model [2] and the Wang model [3] are the former, and the Yang multi-release model [9], the Singh multi-release model [5], and the Tandon multi-release model [6] are the latter. For comparison, we use Case 5 in the literature [5] as the Singh multi-release model, and Model-1 in the literature [6] as the Tandon multi-release model. We use four comparison criteria for the goodness of fit, namely, MSE [10], R^2^ [10], TS [10], and Bias [10], for the fitting performance comparison. We use four comparison criteria for prediction, namely, PSSE [11], TS [10], Variance [10], and Bias [10], for the predictive performance comparison. Table 1 lists model comparison criteria in detail.

**Table 1:** Model comparison criteria for OSS.

Comparion Criteria	Formulation	Description
MSE	Goodness-of-fit: $\frac{\sum_{i=1}^{k_{1}}{\left(X_{t_{i}}-\Lambda (t_{i})\right)}^{2}}{k_{1}}$	where $X_{t_{i}}$ represents the actually observed failure data by time *t*_*i*_. Λ(*t*_*i*_) denotes the expected cumulative number of faults detected by time *t*_*i*_. *k*_1_ is the fitting sample size of fault dataset.
R^2^	Goodness-of-fit: $1-\frac{\sum_{i=1}^{k_{1}}{\left(X_{t_{i}}-\Lambda (t_{i})\right)}^{2}}{\sum_{i=1}^{k_{1}}{\left(X_{t_{i}}-\frac{\sum_{j=1}^{k_{1}}X_{t_{j}}}{k_{1}}\right)}^{2}}$	where $X_{t_{i}}$ represents the actually observed failure data by time *t*_*i*_. Λ(*t*_*i*_) denotes the expected cumulative number of faults detected by time *t*_*i*_. *k*_1_ is the fitting sample size of fault dataset.
TS	Goodness-of-fit: $\sqrt{\frac{\sum_{i=1}^{k_{1}}{\left(X_{t_{i}}-\Lambda (t_{i})\right)}^{2}}{\sum_{i=1}^{k_{1}}X_{t_{i}}^{2}}}\times 100\%$ Prediction: $\sqrt{\frac{\sum_{i=k_{1}+1}^{k}{\left(X_{t_{i}}-\Lambda (t_{i})\right)}^{2}}{\sum_{i=k_{1}+1}^{k}X_{t_{i}}^{2}}}\times 100\%$	where $X_{t_{i}}$ represents the actually observed failure data by time *t*_*i*_. Λ(*t*_*i*_) denotes the expected cumulative number of faults detected by time *t*_*i*_. *k*_1_ is the fitting sample size of fault dataset. *k* is the total sample size of fault dataset. *i* = 1,2,3,…,*k*_1_,…,*k*.
Bias	Goodness-of-fit: $\frac{\sum_{i=1}^{k_{1}}\left|X_{t_{i}}-\Lambda (t_{i})\right|}{k_{1}}$ Prediction: $\frac{\sum_{i=k_{1}+1}^{k}\left|X_{t_{i}}-\Lambda (t_{i})\right|}{k-k_{1}}$	where $X_{t_{i}}$ represents the actually observed failure data by time *t*_*i*_. Λ(*t*_*i*_) denotes the expected cumulative number of faults detected by time *t*_*i*_. *k*_1_ is the fitting sample size of fault dataset. *k* is the total sample size of fault dataset. *i* = 1,2,3,…,*k*_1_,…,*k*. Herein, for the convenience of comparison, Bias adopts the form of absolute value. In other words, Bias calculate the sum of the absolute deviation between the estimated number of detected faults and the actual number of observed faults.
Variance	Goodness-of-fit: $\sqrt{\frac{\sum_{i=1}^{k_{1}}{\left(X_{t_{i}}-\Lambda (t_{i})-Bias\right)}^{2}}{k_{1}-1}}$ Prediction: $\sqrt{\frac{\sum_{i=k_{1}+1}^{k}{\left(X_{t_{i}}-\Lambda (t_{i})-Bias\right)}^{2}}{k-k_{1}-1}}$	where $X_{t_{i}}$ represents the actually observed failure data by time *t*_*i*_. Λ(*t*_*i*_) denotes the expected cumulative number of faults detected by time *t*_*i*_. *k*_1_ is the fitting sample size of fault dataset. *k* is the total sample size of fault dataset. *i* = 1,2,3,…,*k*_1_,…,*k*.
PSSE	Prediction: $\frac{\sum_{i=k_{1}+1}^{k}{\left(X_{t_{i}}-\Lambda (t_{i})\right)}^{2}}{k-k_{1}}$	where $X_{t_{i}}$ represents the actually observed failure data by time *t*_*i*_. Λ(*t*_*i*_) denotes the expected cumulative number of faults detected by time *t*_*i*_. *k*_1_ is the fitting sample size of fault dataset. *k* is the total sample size of fault dataset. *i* = 1,2,3,…,*k*_1_,…,*k*. Herein, PSSE denotes that the average value of the deviation between the actually observed data and the predictive data.

### C. Comparative analysis and discussion on the fitting performance of OSS

We use 100% of fault data sets (DS1) to compare the fitting performance of models. Tables 2–4 show that the proposed model has the best fitting performance among all models. The fitting performance of the proposed model is stable, but other models are unstable. For example, the second is the Singh multi-release model-1 in Table 2, but the second is the Tandon multi-release model-3 in Table 4.

**Table 2:** Comparison of the fitting performance of models using 100% of fault data (DS1-1).

model	Parameter estimation values	MSE	R^2^	TS	Bias
Li model	a=122.03,α=0.13936,A=123.22,N=0.5032	32.87	0.6515	51.37	4.16
Wang model	a=100,b=0.000185,β=1.7812,d=1.8967	62.23	0.3402	70.68	5.89
Yang multi-release model-1	$a_{1}=4514.2,\gamma_{1}=0.000093$	62.12	0.3413	70.62	5.96
Singh multi-release model-1	$a_{1}={82.061,b}_{1}=0.19949,\beta_{1}=1056$	17.39	0.8156	37.36	2.8
Tandon multi-release model-1	$a_{1}={60.159,b}_{1}={0.000001,s}_{1}={3.8684,r}_{1}=0.7039$	26.74	0.7164	46.34	3.44
Proposed model	η=22.194,ψ=0.21739,μ=0.020921,γ=2503.8,C=96.232	13.62	0.8556	33.07	2.4

**Table 3:** Comparison of the fitting performance of models using 100% of fault data (DS1-2).

model	Parameter estimation values	MSE	R^2^	TS	Bias
Li model	a=201.06,α=0.2923,A=237.32,N=0.662	67.44	0.7954	39.13	5.94
Wang model	a=100.01,b=0.000009,β=1.0913,d=2.782	199.81	0.3939	67.35	11.58
Yang multi-release model-2	$a_{2}=4620.6,\gamma_{2}=0.000166$	199.5	0.3948	67.3	11.37
Singh multi-release model-2	$a_{2}={124.43,b}_{2}=0.40675,\beta_{2}=2068.1$	20.38	0.9382	21.51	3.0
Tandon multi-release model-2	$a_{2}={840,b}_{2}={0.000216,s}_{2}={5.0654,r}_{2}=0.0002$	22.03	0.9332	22.36	3.04
Proposed model	η=18.418,ψ=0.44633,μ=0.047692,γ=2502.4,C=117.36	18.62	0.9435	20.56	2.86

**Table 4:** Comparison of the fitting performance of models using 100% of fault data (DS1-3).

model	Parameter estimation values	MSE	R^2^	TS	Bias
Li model	a=97.352,α=0.003807,A=0.15473,N=25.11	202.33	0.6948	38.37	12.67
Wang model	a=100.01,b=0.00001,β=5.282,d=2.8559	129.43	0.8048	30.69	10.11
Yang multi-release model-3	$a_{3}=5957.1,\gamma_{3}=0.000081$	306.75	0.5374	47.24	15.64
Singh multi-release model-3	$a_{3}={789.29,b}_{3}=0.035102,\beta_{3}=103.32$	36.3	0.9452	16.25	4.97
Tandon multi-release model-3	$a_{3}={139.98,b}_{3}={0.1028,s}_{3}={1.8955,r}_{3}=0.0003$	32.31	0.9513	15.33	4.19
Proposed model	η=0.2773,ψ=0.12273,μ=0.016997,γ=303.83,C=74.844	2.33	0.9965	4.12	1.24

Table 2 shows that the last is the Wang model, and MSE of the Wang model is nearly five times as large as that of the proposed model. In Table 3, the last is also the Wang model-2, and its MSE is nearly eleven times as large as that of the proposed model. Table 4 shows that the last is the Yang multi-release model-3, and its MSE is approximately one hundred and fifty-two times larger than that of the proposed model.

**Fig 1:**
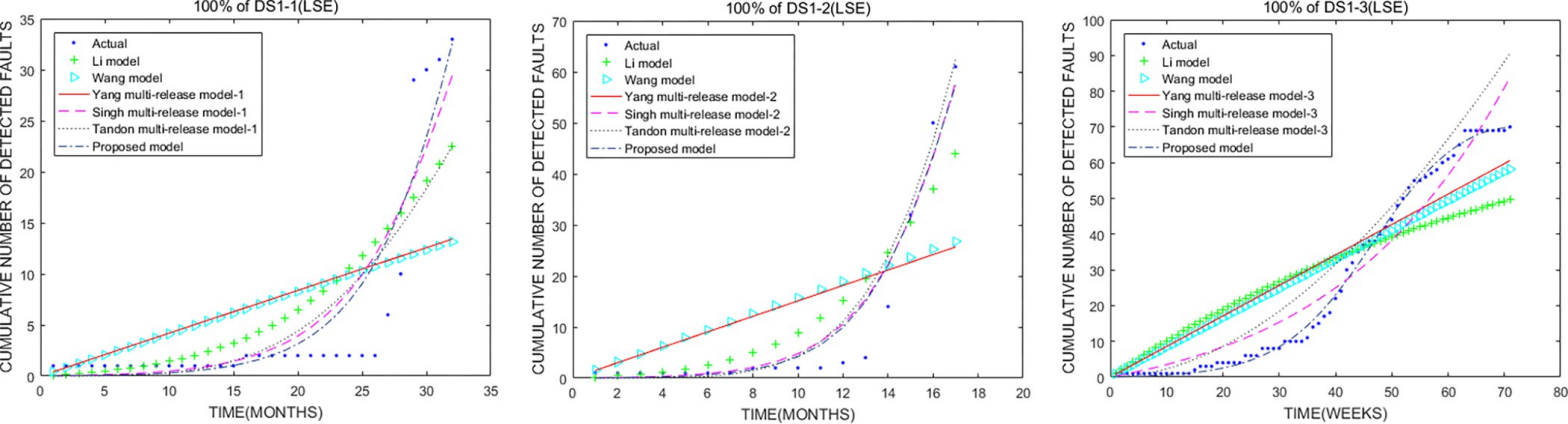
Comparison of the cumulative number of faults estimated by the models using Apache Storm (DS1). (a), (b), and (c) represent the cumulative number comparison of faults estimated by models using 100% of fault data (STORM 1.0.1, STORM 1.0.2, and STORM 1.0.3), respectively.

Overall, the fitting performance of the proposed model is the best among all models. The fitting performance of the Singh multi-release model is equal to that of the Tandon multi-release model, and they are better than other models except the proposed model. In terms of the goodness of fit, the Wang model is equal to the Yang multi-release model, and they are worse than other models. Fig 1(A)–1(C) show the comparison results on the fitting performance of the selected models in this study.

### D. Comparative analysis and discussion on the predictive performance of OSS

**Fig 2:**
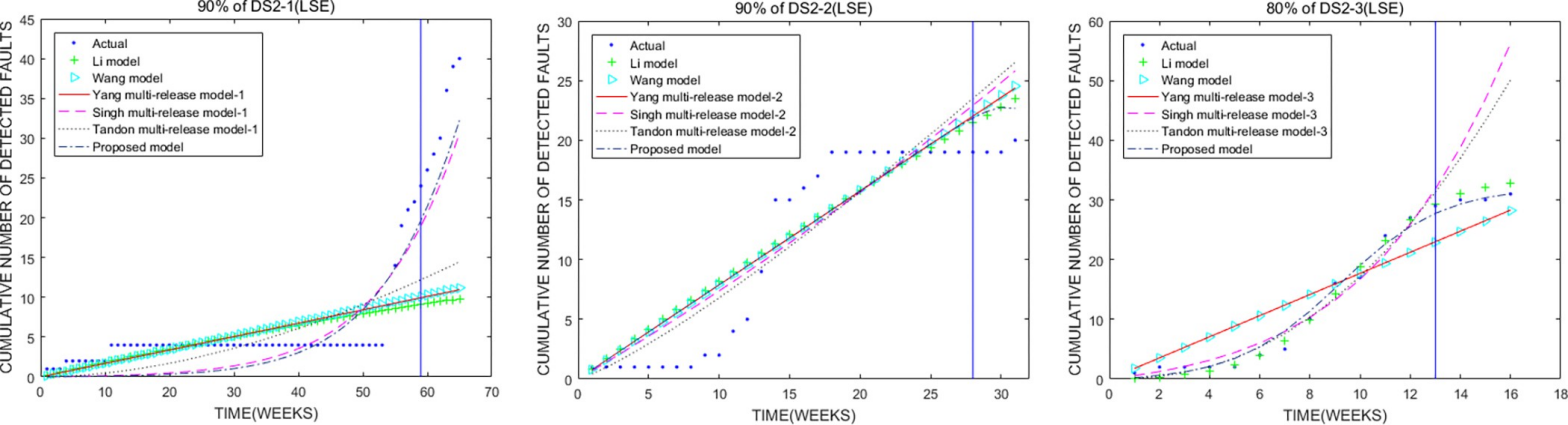
Comparison of the cumulative number of faults estimated by the models using Apache Chemistry OpenCMIS (DS2). (a), (b), and (c) represent the cumulative number comparison of faults estimated by models using 90%, 90%, and 80% of fault data (OpenCMIS 0.4.0, OpenCMIS 0.5.0, and OpenCMIS 0.6.0), respectively.

We use 90% of fault data sets (DS2-1), 90% of fault data sets (DS2-2), and 80% of fault data sets (DS2-3) to randomly select samples for comparing the predictive performance of models. In Table 5, the first is the proposed model, the second is the Singh multi-release model-1, and the last is the Li model based on PSSE, TS, variance, and bias comparisons. PSSE of the proposed model is nearly twelve times as large as that of the proposed model. Table 6 shows that the first is the proposed model, the second is the Li model, and the last is the Tandon multi-release model-2. PSSE of the proposed model is nearly three times less than that of the Tandon multi-release model-2. Table 7 shows that the proposed model has the best predictive performance among all models. The second is the Li model, and the last is the Singh multi-release model-3. PSSE of the proposed model is nearly fourteen times as low as that of the Li model. As shown in Fig 2, the predictive ability the proposed model is better than that of other models.

**Table 5:** Comparison of the predictive performance of models using 90% of fault data (DS2-1).

model	Parameter estimation values	PSSE	TS	Variance	Bias
Li model	a=99.93,α=0.005954,A=0.065531,N=5.4776	586.35	72.05	5.72	23.64
Wang model	a=100.01,b=0.000013,β=1.0931,d=2.2211	529.48	68.47	5.63	22.4
Yang multi-release model-1	$a_{1}=2520,\gamma_{1}=0.000067$	538.89	69.07	5.64	22.64
Singh multi-release model-1	$a_{1}={191.24,b}_{1}=0.091313,\beta_{1}=1978.9$	65.76	24.13	2.4	7.81
Tandon multi-release model-1	$a_{1}={114.87,b}_{1}={0.000085,s}_{1}={1.8604,r}_{1}=0.6478$	409.56	60.22	5.26	19.66
Proposed model	η=1.0014,ψ=0.10575,μ=0.010245,γ=2500.8,C=115.16	46.49	20.29	2.15	6.53

**Table 6:** Comparison of the predictive performance of models using 90% of fault data (DS2-2).

model	Parameter estimation values	PSSE	TS	Variance	Bias
Li model	a=2140,α=0.011634,A=0.23588,N=0.222	11.95	17.88	8.45	3.45
Wang model	a=100.01,b=0.000043,β=99.922,d=3.1348	19.54	22.86	10.81	4.41
Yang multi-release model-2	$a_{2}=1395.6,\gamma_{2}=0.000202$	18.23	22.08	10.44	4.26
Singh multi-release model-2	$a_{2}={179.86,b}_{2}=0.01604,\beta_{2}=6.854$	30.55	28.58	13.5	5.51
Tandon multi-release model-2	$a_{2}={1140,b}_{2}={0.000543,s}_{2}={1.2108,r}_{2}=0.623$	38.46	32.07	15.16	6.18
Proposed model	η=0.0008,ψ=0.002117,μ=0.05299,γ=1.095,C=780.12	10.9	17.07	8.03	3.27

**Table 7:** Comparison of the predictive performance of models using 80% of fault data (DS2-3).

model	Parameter estimation values	PSSE	TS	Variance	Bias
Li model	a=136.36,α=0.55216,A=224.94,N=0.285	2.84	5.56	4.02	1.62
Wang model	a=740.2,b=0.00001,β=0.46541,d=1.774	16.28	13.3	1.29	3.89
Yang multi-release model-3	$a_{3}=7650.6,\gamma_{3}=0.000196$	15.58	13.01	1.3	3.8
Singh multi-release model-3	$a_{3}={98.97,b}_{3}=0.21619,\beta_{3}=113.5$	332.83		60.14	42.37
		16.97			
Tandon multi-release model-3	$a_{3}=740{,b}_{3}={0.00002,s}_{3}={2.2935,r}_{3}=0.6246$	196.99		46.27	32.75
		13.14			
Proposed model	η=1.3175,ψ=0.4812,μ=0.059764,γ=81.99,C=31.287	0.2	1.49	0.64	0.37

In summary, the predictive performance of the proposed model is best among all models. From Tables 5–7, we can see that the proposed model has the best stability among all models. However, the predictive performance of other models is not stable. We can conclude from Tables 2–7 that the proposed model has the best fitting and predictive performances. With the change in OSS development and testing environment, the fitting and predictive performances of other models are uncertain and unstable.

### E. Confidence interval

**Fig 3:**
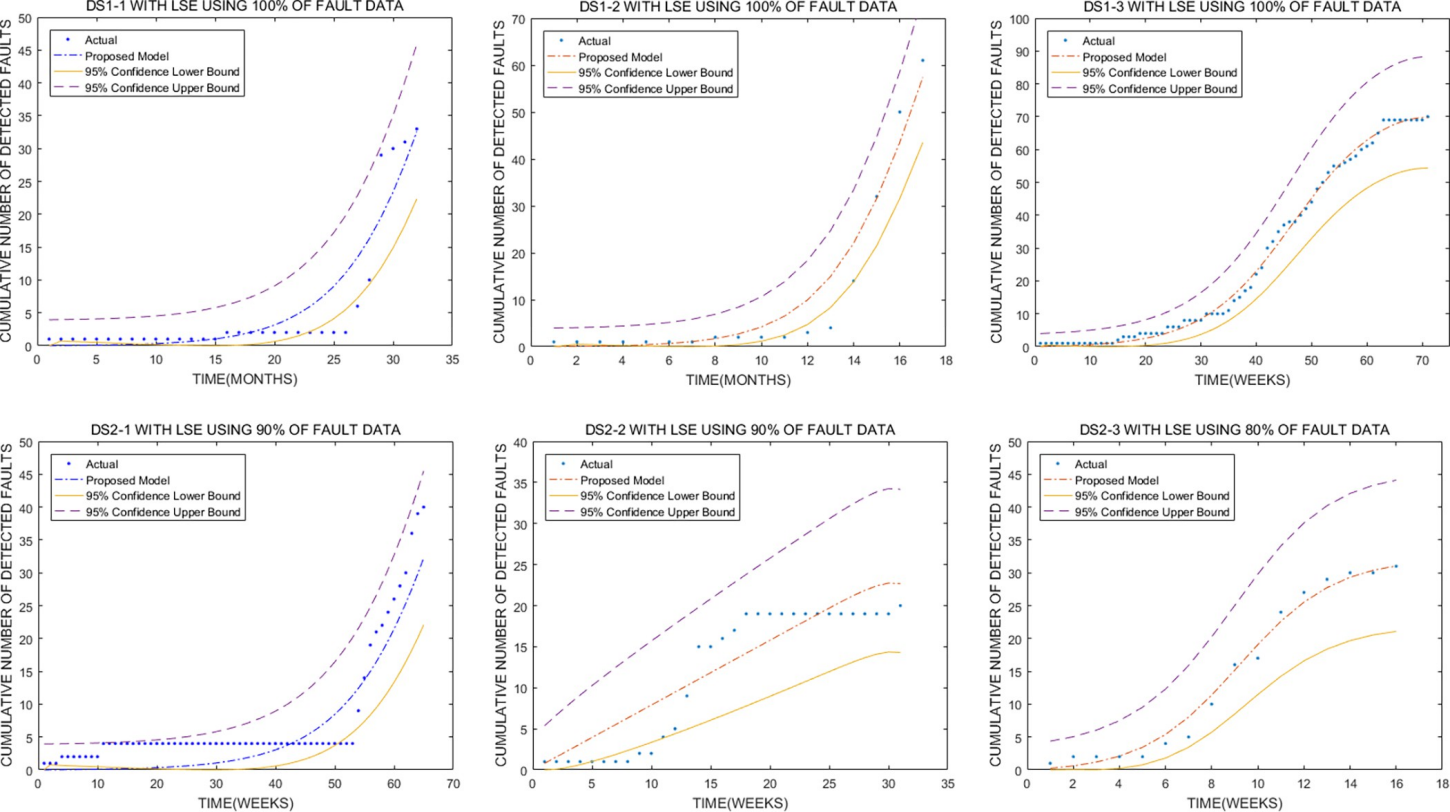
Plots of 95% confidence intervals using fault data (DS1 and DS2). (a), (b), and (c) represent 95% confidence intervals using 100% of fault data (STORM 1.0.1, STORM 1.0.2, and STORM 1.0.3), respectively. (d), (e), and (f) denote 95% confidence intervals using 90%, 90%, and 80% of fault data (OpenCMIS 0.4.0, OpenCMIS 0.5.0, and OpenCMIS 0.6.0), respectively.

Fig 3 shows that most fault data points fall within 95% confidence intervals. However, several fault data points fall outside 95% confidence intervals. For example, Fig 3(A), 3(B), 3(D) and 3(E) show a few fault data points fall outside 95% confidence intervals. Considering the complexity and uncertainty of OSS development and testing, OSS reliability modeling is also quite complex and difficult. Moreover, uncertainty exists in the parameter estimation of the proposed model. Thus, several fault data points will fall outside 95% confidence intervals. Fig 3 shows that most fault data points fall within 95% confidence intervals. These findings indicate that the proposed model has good stability in parameter estimations.

### VI. Sensitivity analysis

**Fig 4:**
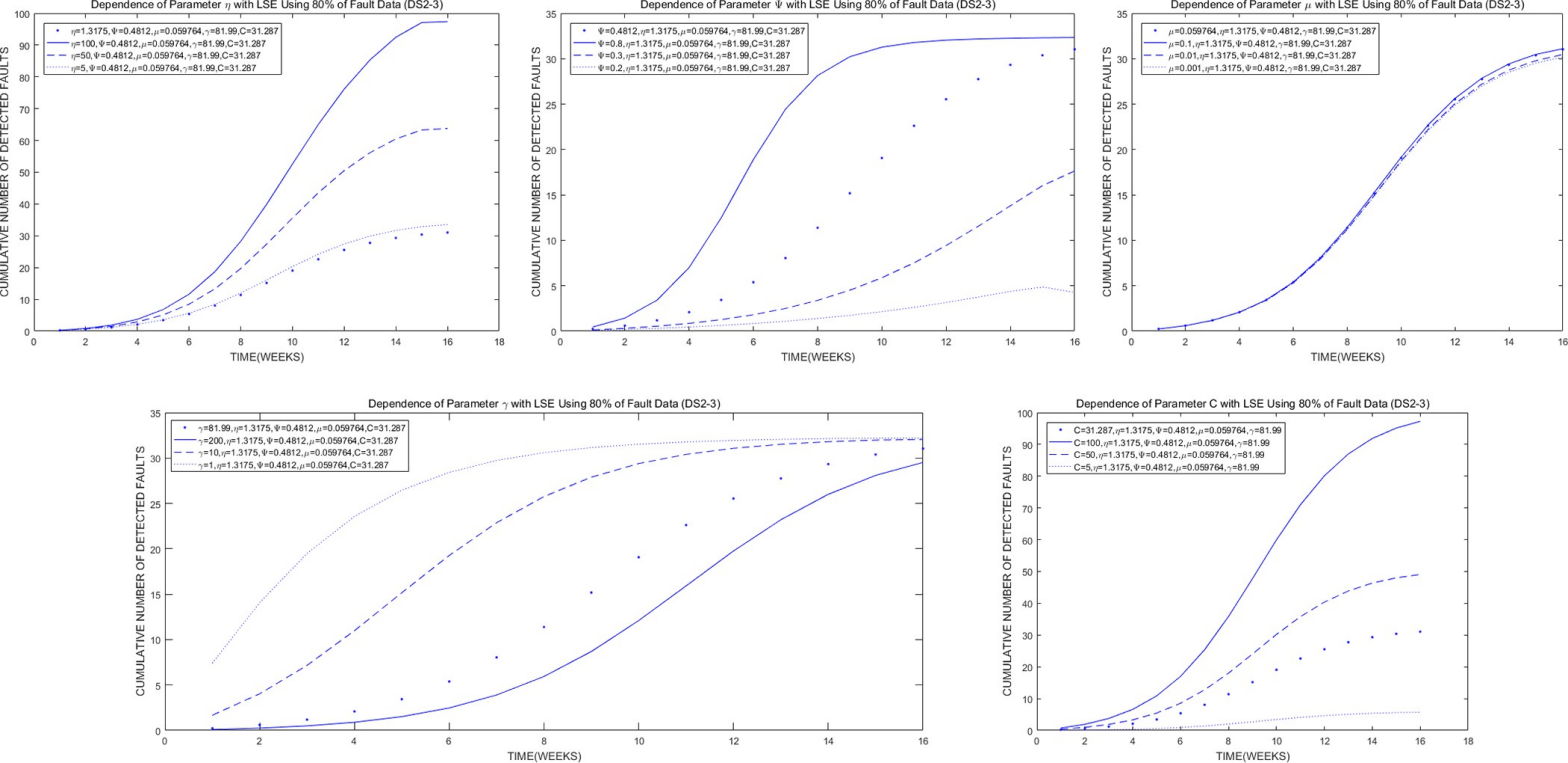
Sensitivity analysis for the parameters of the proposed model. (a) Changes in parameter *η* of the proposed model with LSE using 80% of DS2-3. (b) Changes in parameter *ψ* of the proposed model with LSE using 80% of DS2-3. (c) Changes in parameter *μ* of the proposed model with LSE using 80% of DS2-3. (d) Changes in parameter *γ* of the proposed model with LSE using 80% of DS2-3. (e) Changes in parameter C of the proposed model with LSE using 80% of DS2-3.

We conduct a corresponding sensitivity analysis of model parameters to investigate their important influence in the proposed model. The method of parameter sensitivity analysis facilitates the variation in one parameter and sets other parameter values fixed while studying the parameter change in the model. Fig 4(A), 4(B), 4(D) and 4(E) show that parameters *η*, *ψ*, *γ*, and *C* of the proposed model are important parameters. Thus, the following conclusions can be drawn.

1. In the process of reliability modeling of OSS, fault detection is the key research object. Notably, the change in the fault detection rate (*ψ*) has an important influence on the proposed model.
2. Introduced fault total number (*η*) is also the key research object in establishing a high-quality reliability model of OSS. The change in fault introduction has an important influence on our proposed model.
3. The expected total number of detected faults is also crucial in evaluating the reliability accuracy of OSS. Furthermore, the expected total number of detected faults (*C*) has an important influence on our proposed model.
4. The change in the shape parameter (*γ*) in the proposed model has an important influence on the shape change of the proposed model.

As shown in Fig 4(C), the parameter *μ* of the proposed model is an inactive parameter, that is, a parameter without a significant effect. The reason is that fault introduction is a small probability event compared with fault detection. The shape parameter of the fault detection rate will change because the fault detection rate can vary in many ways. However, the fault introduction rate will be relatively stable and will insignificantly change.

Overall, the establishment of a high-quality reliability model of OSS must consider the changes in fault detection and introduction. Moreover, when the proposed model is used to evaluate the reliability of OSS, we must focus on the changes in the parameters (*η*, *ψ*, *γ*, and *C*) of the proposed model.

## VII. Threats to validity

The threats to validity of the proposed model mainly come from three aspects: threats to internal validity, external validity and construct validity. Three aspects for threats to validity are discussed as follows,

Internal validity: In our study, the threats to internal validity have two influence factors. First, we give an approximate analytical solution to facilitate the use of the proposed model to evaluate software reliability in the actual OSS development and testing process considering the complexity of OSS reliability modeling. Therefore, the performance of the proposed model may be affected. Second, the least square method is used to estimate the parameters’ values of the model, and the parameter estimation is also an approximate value. Considering that the reliability evaluation of OSS is also an approximate probability estimation, the accuracy of our proposed model can meet the requirements.

External validity: In this paper, there are two factors for the threats to external validity. First, few OSS fault data sets and types may be used for experiments, and they may come from the same issue tracking system. However, the fault data sets used in our experiment come from different OSS projects, which fully meets the minimum requirements for model performance comparison. Although the fault comes from the same organization, the fault in the issue tracking system has many attributes and sub-attributes. Therefore, it can meet the basic requirements of software fault types. Second, there may be a few OSS software reliability models we choose to compare, but the selected OSS software reliability models for comparison are the representative.

Construct validity: In order to effectively evaluate and compare the performance of the model, we use six comparison criteria. These comparison criteria can effectively calculate the performance of the model quantitively, and greatly reduce the threats to construct validity. Although we use a few model comparison criteria, they are widely used in software reliability model comparison and evaluation.

## VIII. Related work

In recent years, OSS has been widely used, and its reliability has been the focus of research. The established reliability model of OSS can be divided into the following two categories: perfect debugging (PD) and imperfect debugging (ID).

The PD reliability model of OSS is used when detected faults are removed in the process of OSS testing and no new faults are introduced. For example, Aggarwal et al. [12] proposed an OSS reliability model considering the changing point and fault reduction factor with the Weibull distribution. Kuo et al. [13] proposed an OSS reliability model considering two-parameter generalized Pareto distribution. Huang et al. [14] proposed the new OSS reliability model based on the bounded generalized Pareto distribution through improving the existing OSS reliability models with the generalized Pareto distribution. Tamura and Yamada [15–17] used stochastic differential equation to build the OSS reliability models. Tamura and Yamada [18,19] used deterministic chaos theory to establish the OSS reliability models. Yamada and Tamura [20] proposed a few OSS reliability models considering the influence in OSS modeling. Lee et al. [21] established a prediction model, which is a multivariate linear regression analysis model, by using the OSS life cycle measurement method. Lin and Li [22] proposed a modified queuing theory to establish the corresponding OSS reliability model, which is called rate-based queuing simulation model.

The ID reliability model of OSS is used when the detected faults are removed and new faults may be introduced. For example, Saraf and Iqbal [23] proposed a multi-release ID software reliability model with changing-point. Considering ID and changing-point, Khurshid et al. [24] proposed a multi-release framework of OSS reliability modeling. In addition, Saraf et al. [25] proposed a multi-release software reliability framework not only incorporating ID and change points but also considering fault detection and correction. Considering various levels of faults, Sun and Li [26] proposed the corresponding software reliability model of ID. Considering fault dependency and changing-point, Chatterjee et al. [27] proposed an ID reliability model for multi-upgradation software. In this study, we propose an ID reliability model of OSS considering that fault introduction decreases over time. In the testing process of OSS, fault introduction and detection are complex and random processes. Moreover, fault introduction gradually decreases during OSS testing. Pradhan et al. [28] proposed two ID software reliability models considering the delay between fault detection and introduction and fault decreasing factors with the general inflection S shape. By studying the change- point and ID phenomena during the OSS test, Khurshid et al. [29] put forward a corresponding framework. Considering the different fault severity levels in bug tracking systems, Yanagisawa et al. [30] proposed two kinds of OSS reliability growth models based on a hazard rate. One is a PD software reliability model, and the other is an ID software reliability model.

From the multi-release perspective of OSS, many researchers have established the corresponding OSS reliability models [31,32]. For example, Singh et al. [5] studied the changes of codes and files for OSS during the development and test. They established the corresponding multi-release reliability models of OSS considering using a Cobb–Douglas function to integrate the code changing entropy and testing time. Yang et al. [9] proposed a multi-release reliability framework of OSS considering the delay between fault detection and correction. Garmabaki et al. [33] divided the software testing process into internal and parallel testing. They assumed that faults in current version consist of the remaining faults in the previous version and generated faults due to increasing and modifying components and functions in the current version. Thus, they proposed a multi-release reliability model for OSS. Nijhawan et al. [34] assumed that some errors are added into codes of OSS during up-gradation, and some faults are introduced in the current version besides remnant faults in the previous version. They proposed an ID multi-release reliability model for OSS. Pradhan et al. [35] assumed that fault detection of OSS followed a generalized distribution considering a modified Weibull process, and proposed a multi-release reliability growth model for OSS. Aggarwal et al. [36] considered that the differences in expertise, skills, testing resources, and learning ability of volunteers and users result in a sudden change in the number of detected faults over time. This phenomenon can be called as a changing point on the fault detection rate changes. Therefore, they proposed a multi-release OSS reliability model based on the changing point. Saraf et al. [37] proposed several OSS reliability models considering changing points and ID. Diwakar and Aggarwa [38] considered that faults in the current version comprise two parts. One comprises a few remaining faults in the previous version, and the other is composed of some newly generated faults given that codes, functions, and components are modified in the current version. They proposed a related multi-release reliability model for OSS.

## IX. Conclusions

In this study, we propose an OSS reliability model in which the fault introduction rate decreases over time. Experimental results indicate that the proposed model has better fitting and predictive performances than other models. The parameter sensitivity analysis of the proposed model shows that, except parameter *μ*, other parameters of the proposed model are important parameters. The proposed model can be used for actual OSS reliability evaluation and residual fault prediction. Considering the complexity and uncertainty of OSS development and testing environment, we will deeply study OSS reliability modeling under those environments in the future.

### Appendix A


$$\frac{d\xi (t)}{dt}=\psi (t)(\phi (t)-\xi (t))$$ A.1

ϕ(t)=ηF(t)+C A.2

$$F(t)=1-\frac{\exp(-\mu t)}{1+\mu t}$$ A.3

$$\psi (t)=\frac{\psi }{1+\gamma \exp(-\psi t)}$$ A.4

Given $\varpi (t)=\int_{0}^{t}\psi (x)dx$, then $\exp(\varpi (t))=\frac{\gamma +\exp(\psi t)}{1+\gamma }$.

Both sides of Eq (A.1) are multiplied by exp(*ϖ*(*t*)),

$$\begin{array}{l} \exp(\varpi (t))d\xi (t)+\exp(\varpi (t))\psi (t)\xi (t)=\exp(\varpi (t))\psi (t)\phi (t)dt\\ \int d(\exp(\varpi (t))\xi (t))=\int \exp(\varpi (t))\psi (t)\phi (t)dt\\ \exp(\varpi (t))\xi (t)=\int \phi (t)d(\exp(\varpi (t))) \end{array}$$ A.5

$$\begin{array}{l} \xi (t)=\frac{\phi (t)\exp(\varpi (t))-\int \exp(\varpi (t))d(\phi (t))}{\exp(\varpi (t))}\\ \;=\phi (t)-\frac{1}{\gamma +\exp(\psi t)}\int \left(\gamma +\exp(\psi t)\right)d(\phi (t))\\ \;=\phi (t)-\frac{\gamma \phi (t)}{\gamma +\exp(\psi t)}-\frac{\int \exp(\psi t)d(\phi (t))}{\gamma +\exp(\psi t)}\\ \;=\frac{\phi (t)}{1+\gamma \exp(-\psi t)}-\frac{\eta \mu \int \exp(\psi t‐\mu t)(2+\mu t){\left(1+\mu t\right)}^{-2}dt}{\gamma +\exp(\psi t)} \end{array}$$

$$\begin{array}{l} \xi (t)=\frac{\phi (t)}{1+\gamma \exp(-\psi t)}-\frac{\eta \mu \int \exp(\psi t-\mu t)({\left(1+\mu t\right)}^{-2}+{\left(1+\mu t\right)}^{-1})dt}{\gamma +\exp(\psi t)}\\ \;=\frac{\phi (t)}{1+\gamma \exp(-\psi t)}-\frac{\eta \mu \int \exp(\psi t-\mu t){\left(1+\mu t\right)}^{-2}dt+\eta \mu \int \exp(\psi t-\mu t){\left(1+\mu t\right)}^{-1}dt}{\gamma +\exp(\psi t)} \end{array}$$

$$\xi (t)=\frac{\phi (t)}{1+\gamma \exp(-\psi t)}-\frac{\eta \mu \exp(-\psi t)}{1+\gamma \exp(-\psi t)}(\int \exp(\psi t-\mu t){\left(1+\mu t\right)}^{-2}dt+\int \exp(\psi t-\mu t){\left(1+\mu t\right)}^{-1}dt)$$ A.6

The following expression is extended with Taylor formula,

$$\exp((\psi -\mu )t)=\sum_{i=0}^{n}\frac{{\left(\psi -\mu \right)}^{i}t^{i}}{i!}$$

$$\begin{array}{l} {\left(1+\mu t\right)}^{-2}=1+\frac{-2\mu t}{1!}+\frac{(-2)(-3){\left(\mu t\right)}^{2}}{2!}+,\ldots ,+\frac{{\left(-1\right)}^{m}(m+1)!{\left(\mu t\right)}^{m}}{m!}\\ \;=1-2\mu t+3{\left(\mu t\right)}^{2}+,\ldots ,+{\left(-1\right)}^{m}(m+1){\left(\mu t\right)}^{m} \end{array}$$

$$\begin{array}{l} {\left(1+\mu t\right)}^{-1}=1+\frac{-\mu t}{1!}+\frac{(-1)(-2){\left(\mu t\right)}^{2}}{2!}+,\ldots ,+\frac{{\left(-1\right)}^{m}m!{\left(\mu t\right)}^{m}}{m!}\\ \;=1-\mu t+{\left(\mu t\right)}^{2}+,\ldots ,+{\left(-1\right)}^{m}{\left(\mu t\right)}^{m} \end{array}$$

Then,

$$\begin{array}{l} \int \exp(\psi t-\mu t){\left(1+\mu t\right)}^{-1}dt=\int (\sum_{i=0}^{n}\frac{{\left(\psi -\mu \right)}^{i}t^{i}}{i!}-\mu \sum_{i=0}^{n}\frac{{\left(\psi -\mu \right)}^{i}t^{i+1}}{i!}+\mu^{2}\sum_{i=0}^{n}\frac{{\left(\psi -\mu \right)}^{i}t^{i+2}}{i!}+,\ldots ,+{\left(-1\right)}^{m}\mu^{m}\sum_{i=0}^{n}\frac{{\left(\psi -\mu \right)}^{i}t^{i+m}}{i!})dt\\ \;=\sum_{j=0}^{m}\sum_{i=0}^{n}\frac{{\left(-1\right)}^{j}\mu^{j}{\left(\psi -\mu \right)}^{i}t^{i+j+1}}{(i+j+1)i!}+C1 \end{array}$$

$$\begin{array}{l} \int \exp(\psi t-\mu t){\left(1+\mu t\right)}^{-2}dt=\int (\sum_{i=0}^{n}\frac{{\left(\psi -\mu \right)}^{i}t^{i}}{i!}-2\mu \sum_{i=0}^{n}\frac{{\left(\psi -\mu \right)}^{i}t^{i+1}}{i!}+3\mu^{2}\sum_{i=0}^{n}\frac{{\left(\psi -\mu \right)}^{i}t^{i+2}}{i!}+,...,+{\left(-1\right)}^{m}(m+1)\mu^{m}\sum_{i=0}^{n}\frac{{\left(\psi -\mu \right)}^{i}t^{i+m}}{i!})dt\\ \;=\sum_{j=0}^{m}\sum_{i=0}^{n}\frac{{\left(-1\right)}^{j}(j+1)\mu^{j}{\left(\psi -\mu \right)}^{i}t^{i+j+1}}{(i+j+1)i!}+C2 \end{array}$$

$$\xi (t)=\frac{\phi (t)}{1+\gamma \exp(-\psi t)}-\frac{\eta \mu \exp(-\psi t)}{1+\gamma \exp(-\psi t)}(\sum_{j=0}^{m}\sum_{i=0}^{n}\frac{{\left(-1\right)}^{j}(j+2)\mu^{j}{\left(\psi -\mu \right)}^{i}t^{i+j+1}}{(i+j+1)i!}+C3)$$ A.7

when t = 0, *ζ*(*t*) = 0. Then,

$$C3=\frac{C}{\eta \mu }$$ A.8

Instituting Eqs (*A*.2), (*A*.3), and (*A*.8) into Eq (*A*.7) yields

$$\xi (t)=\frac{\phi (t)}{1+\gamma \exp(-\psi t)}-\frac{\eta \mu \exp(-\psi t)}{1+\gamma \exp(-\psi t)}(\sum_{j=0}^{m}\sum_{i=0}^{n}\frac{{\left(-1\right)}^{j}(j+2)\mu^{j}{\left(\psi -\mu \right)}^{i}t^{i+j+1}}{(i+j+1)i!}+\frac{C}{\eta \mu })$$ A.9

$$\xi (t)=\frac{\eta (1-\frac{\exp(-\mu t)}{1+\mu t})+C(1-\exp(-\psi t))}{1+\gamma \exp(-\psi t)}-\frac{\eta \mu \exp(-\psi t)\sum_{j=0}^{m}\sum_{i=0}^{n}\frac{{\left(-1\right)}^{j}(j+2)\mu^{j}{\left(\psi -\mu \right)}^{i}t^{i+j+1}}{(i+j+1)i!}}{1+\gamma \exp(-\psi t)}$$ A.10

## Supporting information

S1 Data (DOC)
