# Supplementary material for: Software reliability model of open source software based on the decreasing trend of fault introduction
Source: PLoS One. 2022 May 2;17(5):e0267171. doi: 10.1371/journal.pone.0267171 (PMC9060381; doi:10.1371/journal.pone.0267171)
Supplement: S1 Data — (DOC) [file pone.0267171.s001.doc]

| Table 7. OSS fault data sets collected from Apache Storm project(STORM ) (DS1) | | | | | | | |
| --- | --- | --- | --- | --- | --- | --- | --- |
| STORM 1.0.1 (Release-1) | | STORM 1.0.2(Release-2) | | STORM 1.0.3(Release-3) | | | |
| Months | Cumulative number of detected faults | Months | Cumulative number of detected faults | Weeks | Cumulative number of detected faults | Weeks | Cumulative number of detected faults |
| 1  2  3  4  5  6  7  8  9  10  11  12  13  14  15  16  17  18  19  20  21  22  23  24  25  26  27  28  29  30  31  32 | 1  1  1  1  1  1  1  1  1  1  1  1  1  1  1  2  2  2  2  2  2  2  2  2  2  2  6  10  29  30  31  33 | 1  2  3  4  5  6  7  8  9  10  11  12  13  14  15  16  17 | 1  1  1  1  1  1  1  2  2  2  2  3  4  14  32  50  61 | 1  2  3  4  5  6  7  8  9  10  11  12  13  14  15  16  17  18  19  20  21  22  23  24  25  26  27  28  29  30  31  32  33  34  35  36  37  38  39  40  41  42  43  44 | 1  1  1  1  1  1  1  1  1  1  1  1  1  1  2  3  3  3  4  4  4  4  4  6  6  6  8  8  8  8  10  10  10  10  11  14  15  17  18  22  24  30  32  35 | 45  46  47  48  49  50  51  52  53  54  55  56  57  58  59  60  61  62  63  64  65  66  67  68  69  70  71 | 35  37  38  38  40  45  44  48  50  53  55  55  56  58  60  61  62  65  69  69  69  69  69  69  69  69  70 |

| Table 8. OSS fault data sets collected from Apache Chemistry OpenCMIS project(OpenCMIS) (DS2) | | | | | | | |
| --- | --- | --- | --- | --- | --- | --- | --- |
| OpenCMIS 0.4.0(Release-1) | | | | OpenCMIS 0.5.0(Release-2) | | OpenCMIS 0.6.0(Release-3) | |
| Weeks | Cumulative number of detected faults | Weeks | Cumulative number of detected faults | Weeks | Cumulative number of detected faults | Weeks | Cumulative number of detected faults |
| 1  2  3  4  5  6  7  8  9  10  11  12  13  14  15  16  17  18  19  20  21  22  23  24  25  26  27  28  29  30  31  32  33  34  35  36  37  38  39  40  41  42  43  44 | 1  1  1  2  2  2  2  2  2  2  4  4  4  4  4  4  4  4  4  4  4  4  4  4  4  4  4  4  4  4  4  4  4  4  4  4  4  4  4  4  4  4  4  4 | 45  46  47  48  49  50  51  52  53  54  55  56  57  58  59  60  61  62  63  64  65 | 4  4  4  4  4  4  4  4  4  9  14  19  21  22  24  26  28  30  36  39  40 | 1  2  3  4  5  6  7  8  9  10  11  12  13  14  15  16  17  18  19  20  21  22  23  24  25  26  27  28  29  30  31 | 1  1  1  1  1  1  1  1  2  2  4  5  9  15  15  16  17  19  19  19  19  19  19  19  19  19  19  19  19  19  20 | 1  2  3  4  5  6  7  8  9  10  11  12  13  14  15  16 | 1  2  2  2  2  4  5  10  16  17  24  27  29  30  30  31 |
